# Thrombospondin-2 Influences the Proportion of Cartilage and Bone During Fracture Healing

**DOI:** 10.1359/jbmr.090101

**Published:** 2009-01-05

**Authors:** Douglas K Taylor, Jeffrey A Meganck, Shawn Terkhorn, Rajiv Rajani, Amish Naik, Regis J O'Keefe, Steven A Goldstein, Kurt D Hankenson

**Affiliations:** 1 Unit for Laboratory Animal Medicine, University of Michigan Ann Arbor, Michigan, USA; 4 Department of Orthopaedic Surgery, University of Michigan Ann Arbor, Michigan, USA; 5 Department of Biomedical Engineering, University of Michigan Ann Arbor, Michigan, USA; 6 Department of Animal Biology, School of Veterinary Medicine, University of Pennsylvania Philadelphia, Pennsylvania, USA; 7 Department of Orthopaedics, Center for Musculoskeletal Research, University of Rochester Rochester, New York, USA

**Keywords:** fracture, thrombospondin, μCT

## Abstract

Thrombospondin-2 (TSP2) is a matricellular protein with increased expression during growth and regeneration. TSP2-null mice show accelerated dermal wound healing and enhanced bone formation. We hypothesized that bone regeneration would be enhanced in the absence of TSP2. Closed, semistabilized transverse fractures were created in the tibias of wildtype (WT) and TSP2-null mice. The fractures were examined 5, 10, and 20 days after fracture using μCT, histology, immunohistochemistry, quantitative RT-PCR, and torsional mechanical testing. Ten days after fracture, TSP2-null mice showed 30% more bone by μCT and 40% less cartilage by histology. Twenty days after fracture, TSP2-null mice showed reduced bone volume fraction and BMD. Mice were examined 5 days after fracture during the stage of neovascularization and mesenchymal cell influx to determine a cellular explanation for the phenotype. TSP2-null mice showed increased cell proliferation with no difference in apoptosis in the highly cellular fracture callus. Although mature bone and cartilage is minimal 5 days after fracture, TSP2-null mice had reduced expression of collagen IIa and Sox9 (chondrocyte differentiation markers) but increased expression of osteocalcin and osterix (osteoblast differentiation markers). Importantly, TSP2-null mice had a 2-fold increase in vessel density that corresponded with a reduction in vascular endothelial growth factor (VEGF) and Glut-1 (markers of hypoxia inducible factor [HIF]-regulated transcription). Finally, by expressing TSP2 using adenovirus starting 3 days after fracture, chondrogenesis was restored in TSP2-null mice. We hypothesize that TSP2 expressed by cells in the fracture mesenchyme regulates callus vascularization. The increase in vascularity increases tissue oxemia and decreases HIF; thus, undifferentiated cells in the callus develop into osteoblasts rather than chondrocytes. This leads to an alternative strategy for achieving fracture healing with reduced endochondral ossification and enhanced appositional bone formation. Controlling the ratio of cartilage to bone during fracture healing has important implications for expediting healing or promoting regeneration in nonunions.

## INTRODUCTION

Fracture healing is characterized by defined functional and morphological stages. There is initial formation of a hematoma and associated inflammatory cell influx, followed by vascularization and mesenchymal cell expansion, chondrogenic and osteogenic differentiation, endochondral and intramembranous bone formation, and finally remodeling.(1) At the cellular level, after the initial inflammatory phase, processes that influence healing include mesenchymal cell migration, proliferation, differentiation of the cells to either osteoblasts or chondrocytes, and apoptosis of cells within the callus.(1–4) Angiogenesis and mechanical stability are likely crucial components regulating these cellular events and the resultant callus phenotype.(5,6)

In the majority of clinical cases, fractures heal in an uncomplicated manner; however, in severe fractures, and in certain patient populations, such as diabetics and smokers, there is delayed healing and an increased incidence of nonunions. These cases are generally characterized by an increase in chondrogenesis and fibrous tissue development, with a resultant failure to undergo endochondral ossification. Absence of mechanical stability and reduced vascularity are believed to be important predictors of nonunion development and may result in dysregulation of mesenchymal fate.

A wide variety of secreted factors act locally within the healing fracture to regulate vascularity, mesenchymal cell function, and callus development. As examples, vascular endothelial growth factor (VEGF),(5,7) matrix metalloproteinase 9 (MMP9),(8) bone morphogenetic protein 3 (BMP-3),(9) and placental growth factor(10) have all been shown to regulate cell differentiation, callus size, and healing. In addition, extracellular matrix (ECM) proteins, including collagen(11) and osteopontin,(12) have been shown to affect fracture phenotype.

Thrombospondin-2 (TSP2) is another ECM protein that could play a role in regulating fracture healing. TSP2 is a secreted glycoprotein encoded by the *Thbs2* gene and was the second member discovered in the family of five thrombospondin proteins.(13,14) TSP2 is a matricellular protein that modulates cell–matrix interactions and is highly expressed in developing and healing tissues.(15) Mice with a targeted disruption of the *Thbs2* gene (TSP2-null) exhibit a complex phenotype. In the skeleton, TSP2-null mice possess greater cortical bone thickness as a result of an increase in endocortical bone formation.(16) The increase in bone formation is associated with an increase in mesenchymal progenitor (marrow stromal cell) number, as determined by colony forming unit-fibroblast (CFU-F), and stromal cells lacking TSP2 show an increase in proliferation. TSP2-null mice also exhibit atypical bone formation in response to mechanical loading.(17)

In addition to a pronounced bone phenotype, TSP2-null mice show altered soft tissue wound healing(18) and enhanced recovery to ischemic injury of skeletal muscle(19); both occur secondary to an increase in vascularity. TSP2 directly inhibits endothelial cell growth,(20,21) and exogenously delivered TSP2 can regulate angiogenesis in vivo, particularly in association with cancer.(22,23)

Considering the bone phenotype and the response to cutaneous wounding and muscle ischemia in the absence of TSP2, we hypothesized that TSP2-null mice would exhibit accelerated fracture healing. To test this hypothesis, we used the TSP2-null mouse and its coisogenic wildtype mouse in an in vivo model of fracture healing. We show here that the fracture callus in the TSP2-null mouse exhibits greater vascularity and cell proliferation, enhanced intramembranous bone formation, and reduced endochondral ossification compared with WT mice. The absence of TSP2 expedites callus bone formation by altering the differentiation fate of mesenchymal cells, shifting differentiation toward osteoblasts instead of chondrocytes. When TSP2 is delivered to calluses after fracture using adenovirus, a WT chondrogenic phenotype is restored.

## MATERIALS AND METHODS

### Mice

All procedures were approved by the Institutional Animal Care and Use Committee. The mice used had a targeted disruption of the *Thbs2* gene, which encodes the thrombospondin-2 protein (TSP2-null).(24) Coisogenic WT 129/SvJ mice were used for comparison.

### Surgical procedure

We created closed, transverse fractures in both tibias of 63- to 70-day-old mice using methods similar to those described previously by Hiltunen et al.(25) Briefly, mice were anesthetized for all surgical procedures using isoflurane (Aerrane; Baxter, Deerfield, IL, USA), and 0.05 mg/kg of butorphanol tartrate (Torbugesic-SA; Fort Dodge Animal Health, Fort Dodge, IA, USA) analgesic was administered subcutaneously shortly after anesthetic induction. Both legs were prepared for aseptic surgery. Mice were placed in dorsal recumbency on microwaveable heating pads for the duration of anesthesia to maintain normal body temperature. The stifle joint of the right leg was flexed, and a small incision was made just medial to the tibial tuberosity. A 26-gauge hypodermic needle was used to bore a hole in the cortex of the medial aspect of the tibial tuberosity, slightly distal to the stifle joint. A sterile, 0.009-in-diameter, stainless steel pin was inserted into the created hole and inserted down the length of the tibia in the intramedullary canal until resistance was felt, indicating full insertion. This served as an intramedullary pin that would provide stability at the fracture site. This procedure was repeated for the left leg. After pin insertion, the pins were cut to be flush with the cortex, and the skin defect was closed using tissue adhesive (Nexaband; Abbott Laboratories).

Fractures were created in both legs using a custom-made device that uses a sliding weight and guillotine mechanism. This device produces consistent controlled displacement, high-energy impact force sufficient to induce fractures in mouse tibias. Mice were placed in sternal recumbency, and each leg was individually placed in the guillotine and fractured. Whole body radiographs were generated using a microradiography system (Faxitron, Wheeling, IL, USA) to verify pin placement and fracture gap location. Fractures analyzed were typically midshaft, simple, transverse fractures, although occasionally fracture occurred in the distal one third of the tibia. Tape “splints” were placed on both tibias to provide initial rotational stability to the fracture region for the first 48 h.

Mice recovered after the procedure under heat lamps. Moistened food was placed on the cage bottom, and water was provided ad libitum. Mice were typically ambulatory within 1 h after surgery and were observed eating within a few hours. Mice were maintained in a cage with wireless tops to reduce climbing activity. No mortality was observed throughout the course of this study.

### Tissue harvest and preparation

At harvest, all animals were anesthetized with isoflurane gas anesthetic and humanely killed by cervical dislocation. Right tibias were carefully dissected, the intramedullary pins were removed, and the tibias were placed in 4% paraformaldehyde for 24 h, decalcified in formic acid for 12 h, and transferred to 70% ethanol until further processing for histology or immunohistochemistry (IHC). Left tibias were similarly dissected, wrapped in saline-soaked gauze, and placed in storage at −20°C until μCT scanning and torsional mechanical testing could be performed.

### μCT

**FIG. 1:**
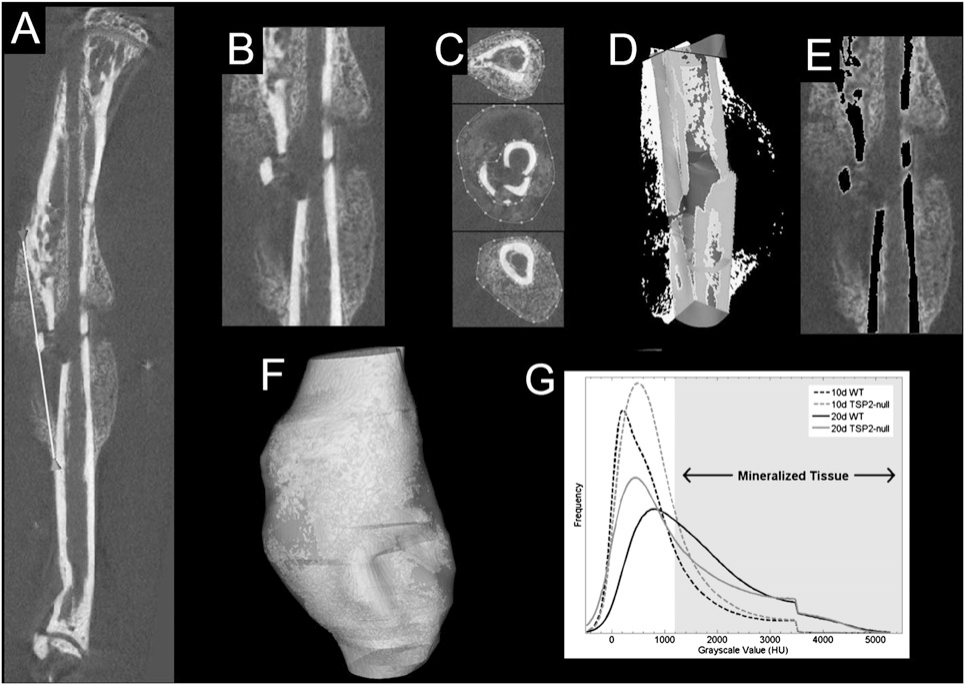
Quantification of callus length, total volume, and bone volume using μCT. (A) Anterior callus length was measured in all specimens (white line). (B) Next, using the maximum callus length, the callus was cropped from the image. (C) Splines were separately traced around the cortical bone and callus. (D) The spline around the cortical bone served as a confining region for a region growing algorithm based on a global threshold (the image shown is binarized at this threshold) that finds all connected cortical bone voxels (the cortical bone that is detected is shown in grey); callus bone is white. (E) The cortical bone voxels are removed from the image by region blanking (gray voxels in (D) becomes black in (E). (F) A final 3D isosurface rendering showing the callus and the region used to analyze the bone that it contains. (G) A histogram of the grayscale values was plotted for callus bone in every specimen. The curves shown are the average of all mice within a particular treatment group and time point. The curve for TSP2-null mice is higher after 10 days, indicating that more bone is present at this time point. After 20 days, the curve for TSP2-null mice is lower, indicating that these mice have less bone in the callus. The shaded region indicates the region used to separate mineralized voxels from unmineralized voxels; the shaded region is used for TMC and TMD calculations, whereas the entire histogram is used for BMC and BMD calculations. The drop-off in the histogram near a grayscale value of 3500 HU is because of the removal of dense bone (which was predominantly residual cortical bone from the original cortices) from the image.

Samples were scanned using an eXplore Locus SP microCT system (GE Healthcare Preclinical Imaging, London, Ontario, Canada) and reconstructed at an 18-μm isotropic voxel size using the Feldkamp cone beam algorithm. A custom software analysis procedure was specifically developed to quantify the callus properties on these images using Microview (v 2.1.2 Advanced Bone Application; GE Healthcare Preclinical Imaging) similar to that described by Den Boer et al.(26) First, the image was reoriented so that the anterior-posterior and longitudinal axes were aligned with the principal image axes. In the second step, three independent reviewers scrolled through the image planes and measured the maximum callus width using a line that bisected the middle of the marrow cavity as well as the maximum callus length on the anterior side of the bone (Fig. 1A). The measurements for maximum callus width and maximum callus length were averaged across the three reviewers, and the average length was used to isolate the callus from the image of the entire bone (Fig. 1B). Next, the callus and cortical bone sections were manually segmented using a series of user-defined points with spline interpolation between these points (Fig. 1C). The points for cortical bone boundary were chosen on slices of the image not more than 30 CT slices (0.540 mm) apart, and spline interpolation was used to define the points in between. These points were reviewed and modified, and a reinterpolation was performed in an iterative process. A similar process was used to define the callus boundary. Next, a single point within the cortical region of interest was used to initiate a region-growing algorithm that detected the cortical bone by finding all connected voxels over a simple global threshold. This region-growing algorithm was confined by the cortical region of interest to ensure that mature bone within the callus, particularly near the proximal and distal ends, was not included in the cortical bone measurements (Fig. 1D). The cortical bone voxels were removed from the image so that it did not bias any measurements (Fig. 1E). Last, the region of interest surrounding the callus was identified (Fig. 1F), a global threshold was applied, and the callus volume, bone volume fraction, BMD, BMC, tissue mineral content (TMC), and tissue mineral density were calculated (TMD). Bone mineral measurements represent the mineral contained in the entire callus volume. Tissue mineral measurements represent the mineral contained within the volume defined as bone (Fig. 1G).

### Mechanical testing

Tibias were secured in brass pots using a low melting temperature Cerro Alloy (McMaster Carr, Chicago, IL, USA) and mounted into a custom torsion testing device. This torsion tester was equipped with a 50 in.oz. reaction torque sensor (Model 2105–50; Eaton, Troy, MI, USA) and an RVDT (Model R30A; Lucas Control Systems, Hampton, VA, USA) for torque and angular displacement measurements, respectively. Raw torque data were conditioned with a strain gage amplifier (2100; Measurements Group, Raleigh, NC, USA), and angular displacement was conditioned with an LVDT amplifier (DTR-451; Lucas Control Systems) before collection. This device was interfaced with LabVIEW (v 7.0; National Instruments, Austin, TX, USA) for data collection and controlled using a custom program that interfaced using a data acquisition system (NI PCI-6251; National Instruments). The bones were tested at a constant displacement rate of 0.5°/s until failure while being maintained moist at room temperature. Data were sampled at 1000 Hz at a displacement rate of 0.5°/s and stored for analysis. Analysis was performed using a custom MATLAB (v 7.0.1; The Mathworks, Natick, MA, USA) script. In this script, the torque data were filtered with a third-order Savitzky-Golay FIR smoothing filter with a 0.5-s window before analysis to remove noise. The stiffness was calculated based on a linear regression on the torque-displacement data in a user-selected region, and the script automated calculations for torque at failure, angular displacement at failure, and energy to failure.

### Safranin-O histology

Safranin-O staining was performed on right mouse tibias that were paraffin embedded and serially sectioned (7 μm). Briefly, slides were deparaffinized, rehydrated, and exposed to 0.3% Fast Green FCF (Fisher, Pittsburgh, PA, USA). Slides were rinsed in 1% acetic acid (Fisher), immersed in 5.45% Safranin-O (Fisher), rinsed in dH_2_O, dehydrated, mounted using Permount (Biomeda, Foster City, CA, USA), and visualized using a microscope. These samples were used for measuring total callus area, chondrocyte area (i.e., Safranin-O–positive area), and area of woven bone. Hypertrophic chondrocyte areas were defined based on the characteristic appearance of those chondrocytes.

### Peroxidase-based IHC

Right mouse tibias were paraffin embedded and serially sectioned (7 μm). Sections were next deparaffinized, rehydrated, immersed in heated citrate buffer, and incubated with 3% H_2_0_2_ in PBS. Slides were blocked and exposed to the respective primary antibody: collagen type IIa (graciously gifted from Dr. Linda Sandell, Washington University), proliferating cell nuclear antigen (PCNA; Chemicon International, Temecula, CA, USA), and von Willebrand's factor (vWF; Dako, Carpinteria, CA, USA). After treatment with the primary antibody (collagen type IIa, 1:5000; PCNA, 1:1500; vWF, 1:400), sections were treated with biotin-conjugated secondary antibody (for collagen type IIa, 1:150; for PCNA, 1:400; for vWF, 1:500; for PCNA: Jackson Immunoresearch, West Grove, PA, USA; for collagen type IIa/vWF: Vector Laboratories, Burlingame, CA, USA). After secondary antibody treatment, slides were treated with streptavidin-conjugated horseradish peroxidase (HRP; StriAviGen Super Sensitive Label Antibody; Biogenex Laboratories, San Ramon, CA, USA) and diaminobenzidine (Dako). Slides were counterstained with Gill's hematoxylin, dehydrated, mounted using Permount (Biomeda, Foster City, CA, USA), and visualized using a microscope. Mouse spleen and heart tissue were used as control tissues for vWF, and mouse testicular tissue was used as positive and negative control tissue for PCNA.

### Fluorescent immunolocalization

Expression of β-galactosidase, osteocalcin (OCN), osterix (OSX), Sox9, TSP2, and VEGFA was assessed by immunofluorescence in serially sectioned (7 μm) paraffin-embedded fractured tibias samples (β-galactosidase/OSX/Sox9; Abcam, Cambridge, MA, USA; OCN: Takara, Shiga, Japan; TSP2: BD Transduction Laboratories, San Jose, CA, USA; VEGFA: Novus, Littleton, CO, USA). After treatment with the primary antibody (β-galactosidase, 1:250; OCN/OSX/Sox9/TSP2/VEGFA, 1:100), sections were treated with Alexafluor 594–labeled secondary antibodies (1:200; Molecular Probes, Carlsbad, CA, USA), mounted with Vectashield containing DAPI (Vector Laboratories, Burlingame, CA, USA), and visualized with a fluorescent microscope.

### TUNEL assay

After tissue rehydration, slides were placed in 95°C citrate buffer for 20 min and, after removal, allowed to cool for 20 min. Next, slides were incubated for 15 min in 3% H_2_O_2_ and incubated for 2 h with the terminal transferase (TdT; Roche, Basel, Switzerland) and biotin-conjugated 16-dUTP (Roche) reaction mixture. Incubation with streptavidin-conjugated HRP and DAB chromogen and hematoxylin counterstain were carried out in the same manner as was performed for IHC. Mouse testicular tissue was used for controls. Positive control tissues were incubated with DNase. Negative controls used testicular tissue without the TdT and or without the 16-dUTP. All control tissues were run in parallel with samples.

### Adenoviral delivery

TSP2 and control β-galactosidase adenovirus were generated by the University of Michigan Vector Core. TSP2-null mice tibias were fractured, and at day 3 after fracture, 10 μl of 1 × 10^8^ TSP2 adenovirus or LacZ control adenovirus particles was injected into the fracture site using Luer Tip Hamilton syringes. Each mouse was injected with LacZ on one side and TSP2 on the contralateral side. The mice were given 10 days to heal after fracture and killed. Tissue was collected and processed as described in the IHC methods and stained according to the Safranin-O protocol detailed earlier.

### Histomorphometry

Total callus area, Safranin-O–positive area, hypertrophic cell area, woven bone area, and the area of cells showing collagen type IIa collagen expression were measured at ×4 magnification using Bioquant Image Analysis software (Bioquant Image Analysis, Nashville, TN, USA). Using the manual measure function of this software, we identified appropriate areas and outlined them. Three tissue sections per slide were measured, and the average was calculated.

For the quantification of PCNA and TUNEL labeling, we used a method similar to that described by Li et al.(2) Briefly, the length of the fracture callus was measured using the Bioquant software at ×4 magnification. Within the proximal, middle, and distal third of the fracture callus, the total number of positive and negative cells was measured in three fields of view at ×63 magnification for a total of nine fields per tissue section. These measurements were repeated on three tissue sections on the same slide for a total of 27 measures per sample. The average number of positive cells over all fields measured represents the percentage of positive cells within the fracture callus.

Total OCN, OSX, Sox9, and VEGFA areas were quantified, at ×200 magnification, using the SigmaScan Pro 5 software (Aspire Software International, Ashburn, VA, USA). Using the manual measure function of this software, positive areas within the total callus were identified and outlined. Three tissue sections per slide were measured, and the average was calculated.

### Time course gene expression analysis for TSP2

WT (C57/B6) mice were placed in groups (*n* = 4 per time point) and given either 0 (no fracture), 5, 7, 10, 14, 18, or 21 days to heal and were subsequently killed. Fracture calluses were carefully dissected and immediately snap frozen in a liquid nitrogen bath. Frozen tissue samples were homogenized using a liquid nitrogen-cooled mortar and pestle apparatus, and mRNA was purified using TRIzol (Invitrogen, Carlsbad, CA, USA). Single-strand cDNA was synthesized from exactly 0.5 μg mRNA from each sample. Specific primers for β-actin (internal control) and TSP2 were used for real-time PCR analysis (Corbett Research, Carlsbad, CA, USA). Samples were denatured at 94°C for 20 s, annealed at 58°C for 30 s, and amplified at 72°C for 30 s for a total of 30 cycles. C(t) results were compared with β-actin expression and fold-change (relative to day 9 nonfracture controls) was determined using previously described methodology.(22)

### QPCR gene expression analysis for day 5 samples

Fracture calluses were extracted and macerated using a Tissue Tearor (Biospec Products, Bartlesville, OK, USA). RNA was isolated using the Qiagen RNeasy Mini kit (Qiagen, Valencia, CA, USA). RNA yield was determined spectrophotometrically, and integrity was confirmed by gel electrophoresis. cDNA synthesis was done as described previously. The expression levels of the following genes were determined using a 7500 Fast Real-Time System machine (Applied Biosystems, Foster City, CA, USA): collagen type IIa, OCN, OSX, Runx2, Sox9, VEGFA, β-actin, and laminA. Taqman Gene Expression Assays (Applied Biosystems) were used for OSX, Runx2, Sox9, VEGFA, and LaminA (internal control), and the following primers were used for collagen type IIa, OCN, and β-actin (internal control) with Power SYBR Green (Applied Biosystems) [Forward (F)/Reverse(R)]:

collagen type IIa: (F)GGCTCCCAGAACATCACCTA/(R)TCGGCCCTCATCTCTACATC
osteocalcin: (F)CGCTCTGTCTCTCTGACCTC/(R)TCACAAGCAGGGTTAAGCTC
β-actin: (F)AAGAGCTATGAGCTGCCTGA/(R)TGGCATAGAGGTCTTTACGG

RNA fold expression levels were calculated using the double ΔCT method, and proper amplicon formation was confirmed by melt curve analysis.

### Statistical analysis

One-way ANOVA was used to assess statistical significance between WT and TSP2-null samples at each time point. Results for the temporal analysis of gene expression were analyzed using one-way ANOVA with Tukey posthoc analysis. Animal numbers for each experiment varied and are indicated in the figure legends.

## RESULTS

### TSP2 is highly expressed in 5-day fracture callus

**FIG. 2:**
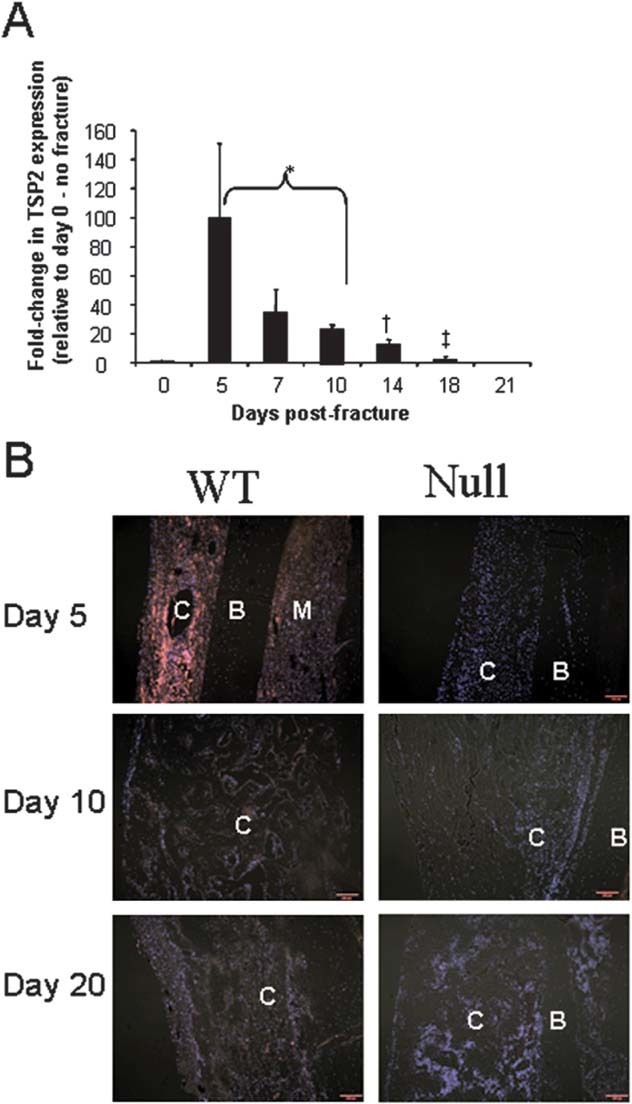
TSP2 expression is increased during early bone regeneration. (A) TSP2 gene expression was characterized at 0, 5, 7, 10, 14, 18, and 21 days after fracture and fold-change in expression was compared with nonfractured bone. *Values are not significantly different (*p* < 0.05) from each other, but are significantly different from all other values, with the exception that the difference between day 14 and day 7 is not significant; ^†^significantly different (*p* < 0.05) from all other values except day 7; ^‡^significantly different from all values with the exception day 0. Bars represent mean and error bars represent SD. *n* = 3. (B) Composite image showing TSP2 detection in vivo using immunofluorescence (magnification bar = 100 μm). Red indicates areas of TSP2 expression, and cell nuclei are shown in blue (B, bone; C, callus; M, marrow).

Previous research has shown that TSP2 is primarily expressed by mesenchymal cells and in mesenchymal origin tissues but is not expressed by hematopoietic cells or endothelial cells.(20,27,28) TSP2 expression was upregulated in response to fracture (Fig. 2A). Levels of TSP2 increased 100-fold in day 5 fractures relative to unfractured day 0 controls and subsequently declined until day 18 when levels normalized. Immunolocalization showed that TSP2 was expressed broadly in the 5-day fractures. TSP2 is expressed highest in the mesenchyme associated with/around the fracture site (Fig. 2B). Fluorescence detection was relatively low in day 10 and day 20 calluses, but a positive signal was present relative to the negative control (TSP2-null).

### TSP2-null mice show alterations in callus morphology

To determine whether an absence of TSP2 would alter fracture healing, we used high-resolution μCT to precisely measure the callus volume, anterior callus length, maximum width, bone volume, mineral content, and mineral density of healing tibial fractures in TSP2-null mice at 10 and 20 days after fracture (dpf) (Table 1). Because of the low bone content of the callus, day 5 fractures could not be evaluated by μCT. Ten days after fracture, callus bone volume, BVF, BMC, BMD, and TMC in TSP2-null mice were significantly greater compared with the callus in WT mice. These data, considered in conjunction with a voxel Hounsfield distribution histogram (Fig. 1G), showed that the TSP2-null mice have more newly formed bone in the callus than WT. Callus shape is also different. Specifically, TSP2-null fractures show a greater callus length to width ratio because of a reduction in length. TMD, which reflects the density of the mineral in voxels identified as bone, was significantly less in the TSP2-null mice than in the WT mice (Table 1). At 20 dpf, the callus volume, width, and width:length ratio were similar between the genotypes. However, callus length was still slightly reduced in the TSP2-null mice. Surprisingly, BVF and BMD were significantly less in the TSP2-null mice after 20 days of healing (Table 1; Fig. 1G).

**Table 1:** μCT Parameters of 10- and 20-Day Harvested Fractures

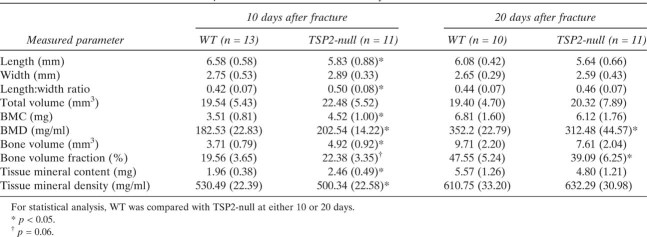

To evaluate the functional consequence of the alterations in bone and callus geometry in the absence of TSP2, tibias were evaluated using torsional testing. Again, because of the low level of bone in the calluses of day 5 fractures, mechanical testing was not performed for these specimens. Despite the increase in bone in the TSP2-null fracture calluses at 10 dpf, there were not any significant differences in the torsional mechanical properties (Table 2). However, in evaluating calluses 20 dpf, the energy to failure of the TSP2-null calluses was significantly decreased.

**Table 2:** Mechanical Properties of Fractures at 10 and 20 Days

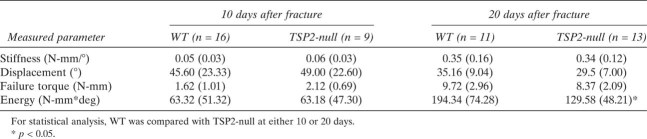

### TSP2-null mice show a reduction in callus cartilage

**FIG. 3:**
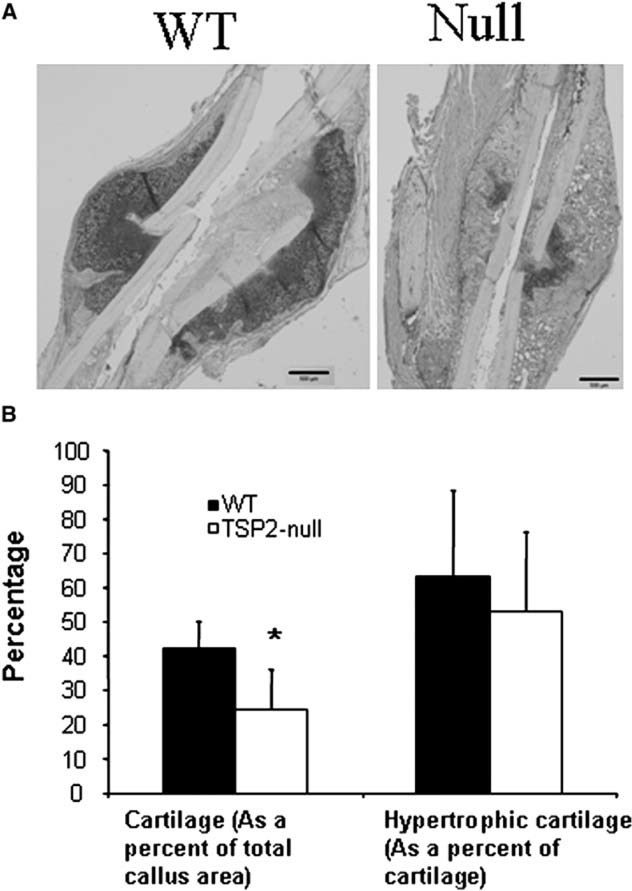
TSP2-null mice have significantly less callus cartilage than WT mice 10 days after fracture. (A) Safranin-O–stained sections of midcallus showing cartilage areas (dark staining) in WT and TSP2-null mice (magnification bar = 500 μm). (B) The percentage of the callus that is cartilage and the percent of cartilage that is hypertrophic was determined using Bioquant image analysis system. Values are mean ± SE of WT (*n* = 14) and TSP2-null (*n* = 13) mice. *Significantly different from WT (*p* < 0.05).

We next evaluated fractures histologically to gain more insight into the alterations in callus geometry and bone content. Qualitatively, there was an obvious reduction in the amount of cartilage in the TSP2-null mice at 10 dpf (Fig. 3A). When we measured the amount of safranin-O–positive cartilage using histomorphometry, the results indicated that fractures in the TSP2-null animals had 40% less cartilage (Fig. 3B). However, there was no difference in the percentage of cartilage that was composed of hypertrophic chondrocytes between the genotypes, suggesting that the progression of cartilage maturation is similar. At day 20, both WT and TSP2-null specimens had very little cartilage. However, even at this time point, all of the WT samples showed some small amount of cartilage, whereas only one half of the TSP2-null specimens contained cartilage (results not shown).

### TSP2-null fractures show differences in callus size and mesenchymal cell proliferation

**FIG. 4:**
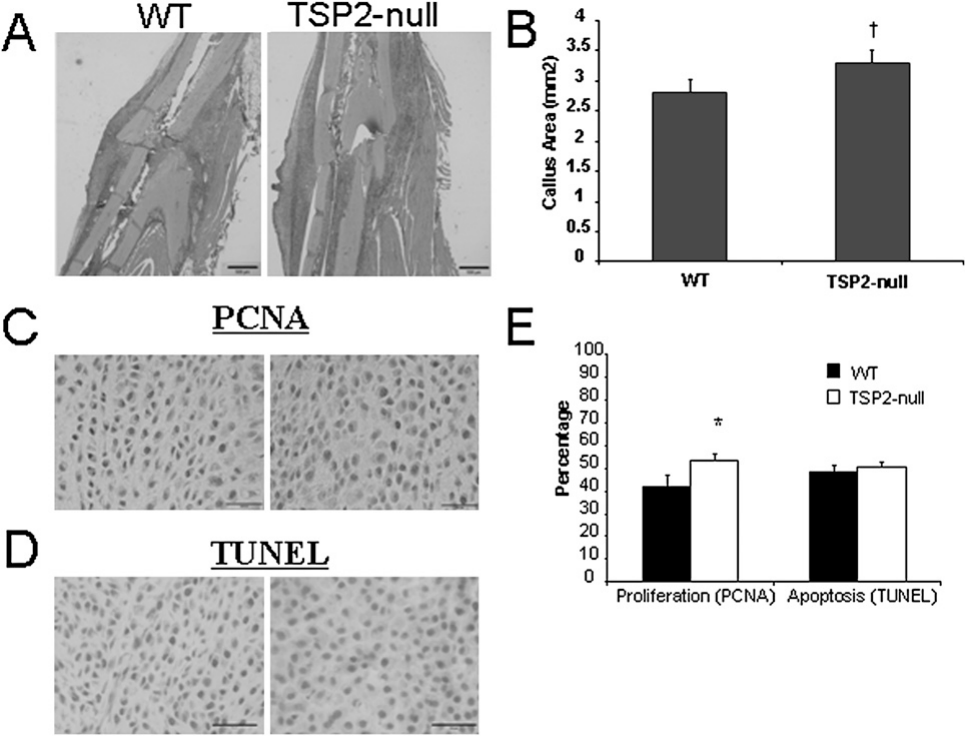
TSP2-null fractures 5 days after fracture show increased callus size and enhanced mesenchymal cell proliferation. (A) Safranin-O–strained tissues showing appearance of provisional callus but an absence of mature cartilage at 5 dpf (magnification bar = 500 μm). Note that Safranin-O staining at this time is generally nonspecific staining of provisional matrix and not bright red relative to the appearance of cartilage in Fig. 3. (B) Area of the fracture callus determined using histomorphometry is slightly greater in TSP2-null mice compared with WT. (C) PCNA expression was examined to evaluate the proliferation of mesenchymal cells. Brown nuclei are PCNA positive (magnification bar = 100 μm). (D) TUNEL was used to evaluate cells undergoing apoptosis. Brown nuclei are TUNEL^+^ (magnification bar = 100 μm). (E) PCNA staining is greater in the fracture callus of TSP2-null mice compared with WT mice, but there is no difference in TUNEL. Values are mean ± SE of WT (*n* = 13) and TSP2-null (*n* = 12) mice. *Significantly different from WT (*p* < 0.05).

Because the fractures of the WT and TSP2-null mice already showed substantial changes by day 10 and we were unable to evaluate day 5 fractures using μCT and mechanical testing, we performed a comprehensive histological and gene expression evaluation of fractures at day 5. The fracture calluses in both the WT and TSP2-null mice at 5 dpf were composed of undifferentiated mesenchymal cells without appreciable bone or cartilage, but TSP2-null fractures had a 20% greater area than WT (Figs. 4A and 4B). Previous work has shown that TSP2 regulates mesenchymal cell proliferation,(16) and additional studies have shown that thrombospondins can regulate apoptosis.(29) Recognizing the importance of these processes in the size of the developing callus, PCNA was used to evaluate mesenchymal cell proliferation, and TUNEL staining was used to evaluate mesenchymal cell apoptosis. Calluses from TSP2-null mice showed a 25% increase in PCNA positive cells compared with those from WT mice (Figs. 4C and 4E), but levels of apoptosis were equivalent (Figs. 4D and 4E).

### TSP2-null mice show decreased chondrogenic differentiation and increased osteoblast differentiation at day 5

**FIG. 5:**
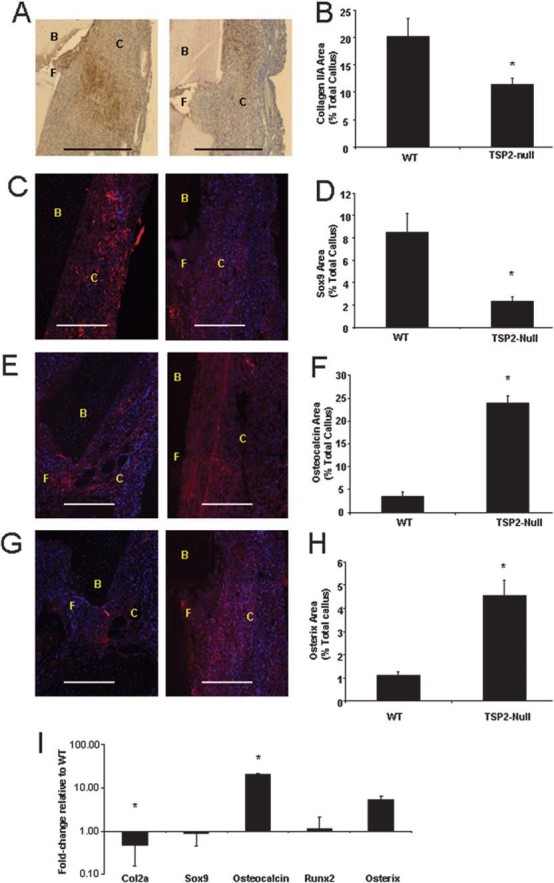
The fracture callus of TSP2-null mice 5 days after fracture has reduced chondrogenesis and increased osteoblast differentiation. Immunohistochemistry and histomorphometry were used to examine the differentiation of mesenchymal cells in the 5d calluses. (A and B) Type IIa collagen and (C and D) Sox9 expression were evaluated to determine areas of neochondrogenesis, whereas (E and F) osteocalcin and (G and H) osterix expression were used to indicate areas of new bone formation (magnification bar = 100 μm). Positive staining is either brown (A) or red (C, E, and G). Blue staining in C, E, and G represents DAPI-stained nuclei. B, bone; C, callus tissue; F, fracture. Values are mean ± SE of WT (*n* = 13) and TSP2-null (*n* = 12) mice. *Significantly different from WT; *p* < 0.05. (I) RNA was extracted from day 5 calluses and gene expression was evaluated using quantitative real-time PCR. Values are mean ± SE of fold-change in TSP2-null (*n* = 6) compared with WT mice (*n* = 5); *ΔCT significantly different from WT (*p* < 0.05).

There is no safranin-O–positive mature cartilage at 5 dpf (Fig. 4A), but mesenchymal cells are beginning to undergo chondrogenic differentiation. Using IHC, we measured the amount of collagen type IIa and Sox9 expression as indicators of early chondrocyte differentiation. Both type IIa collagen and Sox9 expression peak around 5 dpf and diminish with cartilage maturation and hypertrophy.(30,31) The TSP2-null mice had significantly less type IIa collagen–positive area (Figs. 5A and 5B) and reduced expression of Sox9 (Figs. 5C and 5D). To examine osteoblast differentiation of the callus mesenchymal cells, we evaluated osteocalcin and osterix expression. Expression of both osteocalcin (Figs. 5E and 5F) and osterix was significantly increased in TSP2-null fractures (Figs. 5G and 5H). We also harvested total RNA from day 5 fractures and found that the expression of osteocalcin RNA was significantly increased in the TSP2-null mice, whereas type II collagen RNA was significantly decreased (Fig. 5I). These data show that TSP2-null mice have a reduction in chondrogenic differentiation and enhanced osteoblast differentiation.

### TSP2-null fractures show enhanced vascularity and a reduction in markers of hypoxia-inducible factor activity

**FIG. 6:**
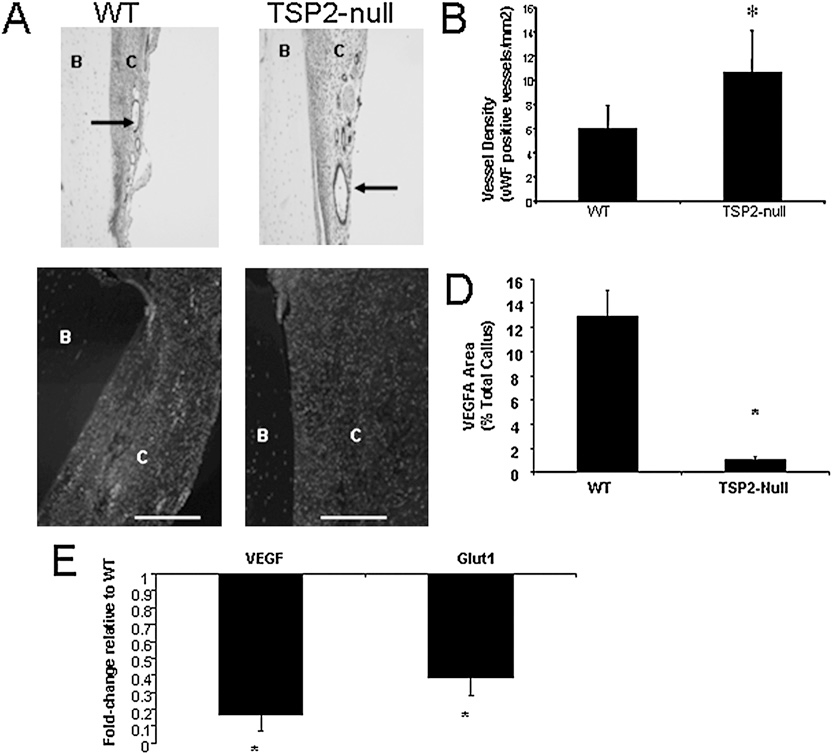
TSP2-null fractures show increased angiogenesis and a reduction in markers associated with HIF1-α activity. (A and B) Blood vessel density was determined by counting the total number of vWF-positive vessels in the callus (arrows). (B) Blood vessel density is 75% greater in the callus of TSP2-null mice compared with WT mice. (C and D) VEGF expression in calluses was evaluated using immunofluorescence (magnification bar = 100 μm). B, bone; C, callus tissue; F, fracture. Values are mean ± SE of WT (*n* = 13) and TSP2-null (*n* = 12) mice. *Significantly different from WT (*p* < 0.05). (E) RNA was extracted from day 5 calluses and gene expression was evaluated using quantitative real-time PCR. Values are mean ± SE of fold-change in TSP2-null (*n* = 6) compared with WT mice (*n* = 5). *ΔCT significantly different from WT (*p* < 0.05).

Thrombospondins are potent regulators of angiogenesis, and previous work has shown that TSP2-null mice show accelerated dermal wound healing(18) and enhanced recovery to ischemia in muscle,(19) caused in part by enhanced vascularity. Because the development of vascularity in the callus is an important part of the fracture healing response, vWF expression within the callus was used to determine vessel density. Importantly, the number of whole blood vessels within the callus that could be identified by their labeling with anti-vWF antibody and the presence of a distinct lumen was 2-fold greater in the TSP2-null mice compared with the WT mice at 5 dpf (Figs. 6A and 6B).

Because hypoxia-inducible factor (HIF) activity is recognized positive regulator of chondrogenic differentiation,(32) we hypothesized that the increased vascularity in TSP2-null mice would result in reduced hypoxia and lead to a reduction in HIF activity. As a surrogate of HIF activity,(33) we evaluated VEGFA expression using IHC and quantitative RT-PCR and Glut-1 expression using quantitative RT-PCR. VEGF expression was reduced in tissue sections (Figs. 6C and 6D), and both Glut-1 and VEGFA were decreased in TSP2-null fractures as measured by qPCR (Fig. 6E).

### Delivery of TSP2 adenovirus to TSP2-null fractures increases chondrogenesis in 10-day fractures

**FIG. 7:**
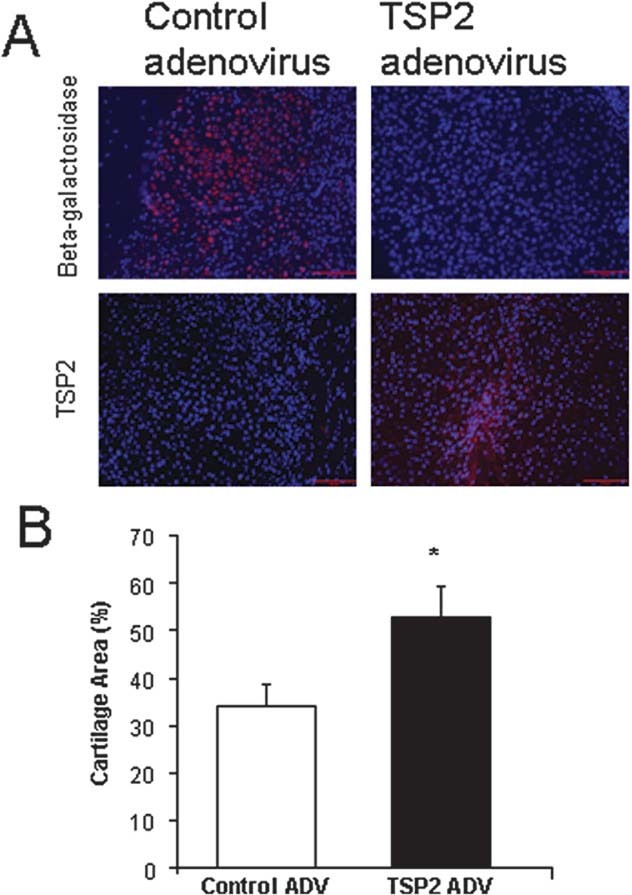
Delivery of TSP2 to TSP2-null mice promotes endochondral bone formation. (A) TSP2 was delivered locally, unilaterally to fractured tibia using adenovirus at 3 days after fracture. The opposite limb received a LacZ control virus. TSP2 or β-galactosidase expression was determined using immunofluorescence (red staining; magnification bar = 100 μm). Blue staining is DAPI^+^ nuclei. (B) Cartilage content was measured using histomorphometry at day 10. Values are mean ± SE of control (*n* = 7) and TSP2-null + TSP2 ADV (*n* = 7) mice. *Significantly different from control (*p* < 0.05).

To examine the temporal requirement of TSP2 to influence fracture cell fate, TSP2 was delivered to fractures in TSP2-null mice 3 dpf during the time of mesenchymal cell influx and neovascularization, and fractures were evaluated using histology at day 10. Adenovirus delivered in this manner was effectively taken up by the cells of the mesenchymal callus (Fig. 7A). TSP2 overexpression resulted in enhanced callus cartilage in TSP2-null mice (Fig. 7B).

## DISCUSSION

Previous work has shown that TSP2 acts to regulates the time course of dermal wound healing(18) and expedites recovery to muscle ischemia.(19) In this study, we found that TSP2 alters the temporal progression of bone regeneration. Ten days after fracture, TSP2-null mice have substantially less cartilage and an increase in the amount of bone. At day 5 when chondrogenic differentiation is just beginning, but there is no mature cartilage, chondrocyte markers are reduced and osteoblast markers are increased in TSP2-null mice. Thus, whereas undifferentiated mesenchymal cells in the central portion of the callus are undergoing differentiation to chondrocytes in WT mice, in TSP2-null mice, fewer cells become chondrocytes and more become osteoblasts. Importantly, the ratio of total cartilage that is hypertrophic (mature) cartilage in WT and TSP2-null mice is equivalent at day 10, suggesting that chondrogenic maturation is normal in the callus of TSP2-null mice.

In the fracture gap, undifferentiated mesenchymal cells that contribute to healing are derived from periosteum, endosteum, surrounding fascia and muscle, and marrow. These mesenchymal cells differentiate to become either osteoblasts or chondrocytes or they undergo apoptosis. The osteoblasts participate in direct intramembranous bone formation, whereas the chondrocytes form cartilage that will subsequently undergo endochondral bone formation.

The molecular mechanisms dictating the fate decision of callus mesenchyme in regenerating bone remains unclear. Whereas a variety of possible factors may contribute to this fate decision, vascularity and oxygen tension in the fracture gap likely plays a prominent role. Hypoxia and the resultant induction of Hif1α has been shown to be prochondrogenic.(32,34,35) Indeed, in our study, we concluded that the dominant mechanistic explanation for the shift in mesenchymal cell fate in WT and TSP2-null mice is that a substantial increase in vascular density in TSP2-null mice alters tissue oxygen tension. Enhanced oxemia promotes the differentiation of bipotent cells to become osteoblasts rather than chondrocytes.

Thrombospondins have been extensively studied as endogenous inhibitors of angiogenesis for 20 yr.(36–38) Mice lacking TSP1 and TSP2 show increases in angiogenesis,(24) and overexpression of TSP prevents tumorigenesis in a number of tissues.(39) Furthermore, in wound healing(18) and tissue ischemia,(19) an absence of TSP2 alters healing through an increase in vascularity. Although TSP1 and TSP2 show considerable homology, data suggest that the TSP1 and TSP2 effects on endothelial cells is different. Whereas TSP1 requires binding to CD36 and activation of downstream kinases to induce apoptosis,(40) TSP2 seems to regulate endothelial cell proliferation through a non–CD36-meditated mechanism that requires VLDLR.(20,21)

A variety of earlier studies have shown a link between angiogenesis and fracture healing. Inhibition of angiogenesis has been shown to cause a decrease in callus mineralization and callus volume.(5,41) In contrast, treatment of fracture calluses with an adenoviral vector carrying a construct encoding VEGFA leading to more vascularity resulted in a reduced amount of cartilage at 2 wk after fracture(42)—a phenotype similar to that seen with TSP2-null mice. Indeed, levels of the direct transcriptional targets of Hif, VEGFA, and Glut-1(33) were significantly reduced in TSP2-null mice.

It is likely that an increase in Hif1α in oxygen-depleted tissues impacts chondrogenic and osteogenic differentiation. Increased activation of HIF1α increases the differentiation of mesenchymal progenitors to chondrocytes,(32,43) and Hif1 directly regulates Sox9 activity.(44) Conversely, increased HIF decreases markers of osteoblast differentiation in vitro.(45) Thus, in the absence of TSP2 when there is higher vascularity, oxygen tension is increased, and Hif1α levels are reduced, favoring osteogenesis.

Although alterations in vascularization and oxemia offer a reasonable explanation for the TSP2-null fracture callus phenotype, an absence of TSP2 could be influencing fracture phenotype through at least two other mechanisms. Increased proliferation of mesenchymal cells in the callus could have a negative influence on chondrogenic differentiation because, to undergo chondrogenic differentiation, cells must first exit the cell cycle.(46–48) It is likely that TSP2-null cell proliferation does account for the 20% increase in callus size at day 5 and the alteration in the width-length ratio observed at day 10. Second, it is possible that the mesenchymal cells that are recruited into the fracture site of the TSP2-null mice are phenotypically different than the cells of the WT. For example, TSP2-null mice have an increase in marrow CFU-F(16); thus, marrow could make a greater contribution to the healing in TSP2-null than WT mice. These alternative explanations can not be ruled out at this time and need to be further studied in models where proliferation of cells is manipulated and by studying earlier time points after fracture in which cell origin can be better discerned.

Surprisingly, despite the significant alterations in callus bone and cartilage after 10 days of healing, we did not detect any changes in the biomechanical properties. Because the biomechanical properties of the healing construct can be attributed both to density and to the amount of tissue, it is plausible that the biomechanical advantage of a higher volume of bone in TSP2-null mice is offset by the decrease in TMD at 10 days. In contrast to these results, the energy to failure was significantly lower in TSP2-null mice after 20 days of healing, whereas the TMD was not different. These results are likely attributable to the decrease in the volume fraction of bone present in the calluses of TSP2-null mice at 20 days. Interestingly, this may suggest that a florid chondrogenic response provides for a mechanically advantageous callus.

The high expression of TSP2 early in fracture healing (Fig. 2) and the phenotype of TSP2-null fractures suggests that TSP2 plays an important role in the regulation of early fracture mesenchyme. Multiple factors must act in concert to influence the fate of early callus mesenchyme. By regulating the balance of proangiogenic factors and factors that regulate mesenchymal cell proliferation and differentiation, we may be able to better influence fracture healing clinically. Indeed, as proof-of-principle, by delivering TSP2 adenovirus, we were effectively able to generate a more chondrogenic fracture callus.
